# Evaluation of an Oral Fluid Collection Device and a Solid-Phase Extraction Method for the Determination of Coca Leaf Alkaloids by Gas Chromatography–Mass Spectrometry

**DOI:** 10.3390/molecules29030592

**Published:** 2024-01-25

**Authors:** Pamela Cabarcos-Fernández, Ivan Álvarez-Freire, Nelida Cristina Rubio, Ana Maria Bermejo-Barrera, Antonio Moreda-Piñeiro, Ines Sánchez-Sellero, Maria Jesus Tabernero-Duque

**Affiliations:** 1 Forensic Toxicology Service, Forensic Sciences Institute, Faculty of Medicine, Universidade de Santiago de Compostela, Rúa de San Francisco, s/n, 15782 Santiago de Compostela, Spain; pamela.cabarcos@usc.es (P.C.-F.); ivan.alvarez@usc.es (I.Á.-F.); cristinarubio2@gmail.com (N.C.R.); anamaria.bermejo@usc.es (A.M.B.-B.); ines.sanchez.sellero@usc.es (I.S.-S.); 2 Trace Element, Spectroscopy and Speciation Group (GETEE), Institute of Materials iMATUS, Department of Analytical Chemistry, Nutrition and Bromatology, Faculty of Chemistry, Universidade de Santiago de Compostela, Avenida das Ciencias, s/n, 15782 Santiago de Compostela, Spain; antonio.moreda@usc.es

**Keywords:** hygrine, cuscohygrine, Quantisal^®^ oral fluid collection device, matrix effect, solid-phase extraction, oral fluid

## Abstract

Some South American countries have ancient traditions that may pose legal problems, such as the consumption of coca leaves, as this can provide positive results for cocaine use after the analysis of biological samples. For this reason, it is necessary to find specific markers that help differentiate legal from illegal consumption, such as tropacocaine, cinnamoylcocaine, and especially hygrine and cuscohygrine. In this work, two techniques for collecting biological samples are compared: the Quantisal^®^ Oral Fluid collection device and passive drooling. Once the samples were collected, they were subjected to solid-phase extraction for subsequent injection into GC-MS. Different validation parameters included in international guides have been studied to evaluate whether the proposed method is valid for the defined purpose, placing special emphasis on the study of the matrix effect and little value on GC-MS analyses. With respect to this parameter, an increase in the signal was found for CUS and t-CIN, but it was not significant for the rest of the substances studied. The recoveries have varied significantly depending on the way of working, being higher when working with standardized areas. After carrying out work with the oral fluid samples collected from laboratory volunteers, the method was applied to two real samples. The results obtained support the need for further research to overcome certain limitations presented by the device.

## 1. Introduction

**Figure 1:**
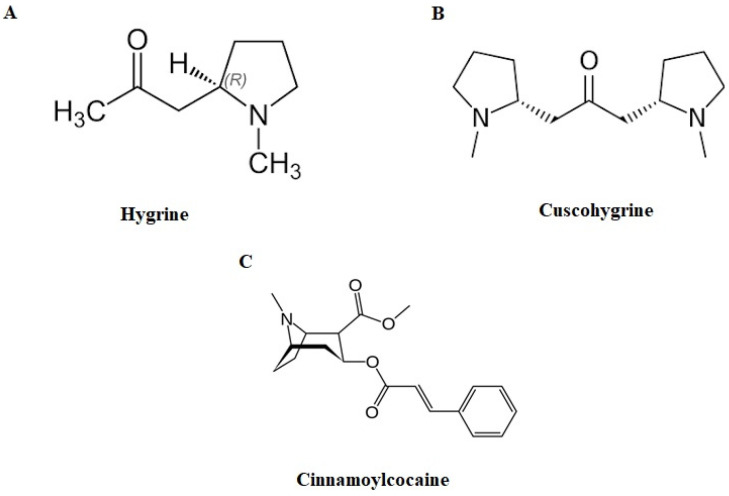
Molecular structures of hygrine (**A**), cuscohygrine (**B**), and cinnamoylcocaine (**C**).

The consumption of coca leaves is a traditional practice (consumed by either being chewed or via drinking coca tea infusions) that generates legal problems in some Latin American countries such as Argentina, Perú, and Bolivia. It is a practice that has not been scientifically proven to cause any harm. The use of coca leaf is permitted by law in Argentina [1] and the Bolivian legal system recognizes its ancestral character, which dates back centuries. In Peru, its cultivation and sale are also permitted. However, the use of the coca leaf remains controversial in many Latin American countries despite many of them having signed the Single Convention on Narcotic Drugs (1961), article 49 of which stipulates the prohibition of chewing coca leaves. This creates the need to determine whether the presence of cocaine in different biological matrices (blood, urine, oral fluid, or hair) is due to legal or illegal use. For some years now, it has been reported that the analysis of coca leaf alkaloids such as hygrine (HYG), cuscohygrine (CUS), tropacocaine (TRO), and cinnamoylcocaine (t-CIN) [2,3,4] are useful to establish whether an individual has consumed coca leaves. HYG and CUS are pyrrolidine alkaloids (Figure 1) present in the coca plant. Both compounds are lost in the first step of illegal cocaine production, so they can be considered markers of legal consumption [5]. Cinnamoylcocaine, a tropane-derived alkaloid with physicochemical characteristics similar to cocaine (logP, pKa, etc.) (Figure 1), accompanies cocaine as a contaminant in its extraction. It must be removed in illegal production by the addition of oxidants, but this step is not always mandatory in clandestine production, so it is not as specific a marker as hygrin and cuscohygrin, which is why it has been considered as a secondary marker. Tropacocaine is found in coca plants in very low concentrations. It is considered a potential marker because it has not yet been reported in illegal cocaine users. The use of the coca leaf to mask illicit cocaine consumption can be suspected by determining, in addition to the alkaloids of the coca leaf, possible adulterants of illegal cocaine and analyzing other matrices such as hair [3]. CUS, HYG, TRO, and t-CIN are not included in routine screening in toxicology laboratories worldwide. In fact, the low relevance currently given to these compounds in toxicology laboratories is demonstrated by the confirmation that, at the time of this study, only the commercial CUS standard was available.

Oral Fluid (OF) is an alternative matrix in forensic toxicology. It is a simple, rapid, accessible, non-invasive, and useful biological sample applicable in the field of forensic toxicology, in criminal justice, workplace testing, and driving under the influence of drugs (DUID) programs [6,7]. However, the results obtained can be affected by factors such as sample pH, drug pKa, protein binding, and sampling. Oral fluid sampling with collection devices has gained great interest in recent years because these biological samples collected by passive spit or drooling are unpleasant for donors and collectors. Additionally, they are less hygienic and difficult to handle when oral fluid samples are taken out of the laboratory environment for, for example, driving controls or workplace testing. However, the oral fluid samples obtained by drooling are of better quality than those obtained using commercial devices because they contain fewer contaminants from the devices. There are several commercial oral fluid collection devices. The Quantisal^®^ oral fluid collection device was chosen for this study due to some of its features. Its has been used in several scientific studies for detecting cocaine and its metabolite benzoylecgonine, with the device achieving good results [8,9]. This device meets the European Workplace Drug Testing Society (EWDT) [10] and The Substance Abuse and Mental Health Services Administration (SHAMSA) [11] requirements as it has an adequate volume indicator (blue dye) and can be used to collect a minimum of 1.0 ± 0.1 mL. In addition, the study laboratory had sufficient stock for this work, as this device is the one commonly used by our laboratory colleagues.

The present work aimed to develop a sensitive oral fluid method that is able to simultaneously determine several coca leaf alkaloids, such as HYG, CUS, TRO, t-CIN, and EME, as well as a metabolite of cocaine and COC, using two ways of sample collection (the Quantisal^®^ oral fluid device and spitting). Solid-phase extraction was used as an extraction technique to concentrate the samples, and gas chromatography–mass spectrometry (GC-EI/MS) was used as a detection technique. This method will be useful for workplace testing and driving under the influence (DUID) programs in Latin American countries. Therefore, the method should meet the requirements set out in the international oral fluid guidelines in terms of cut-off values for COCs; however, there is no specification for other coca alkaloids [11,12]. The second objective of this work was to provide a better understanding of the matrix effect (ME) phenomenon and how it affects the consistency and reliability of experimental results.

As indicated by Peter et al., (2017) [13], once the purpose of the method (separation/detection system) is defined, any other published methods for the same purpose or similar purposes must be identified. To date, no published works have used the Quantisal^®^ device or passive drooling as a sample collection technique together with solid-phase extraction as an extraction technique for the analysis of coca alkaloids. Furthermore, as already mentioned, the coca leaf alkaloids investigated in this work (HYG, CUS, CIN, TRO) are rarely analyzed in forensic toxicology laboratories around the world.

## 2. Results

### 2.1. Experimental Part 1: Matrix Effect (ME)

Table 1 and Table 2 shows that in both samples, the ME caused an enhancement in the signal with some suppression exceptions but with values close to zero. CUS and t-CIN showed a high ME when the samples were collected via drooling and using the commercial Quantisal^®^ device. For example, the ME enhancements for CUS were 576% and 130% at concentrations of 20 ng/mL (A) and 10 ng/mL (B), respectively. Whereas CIN showed percentages of 80% and 46% at concentrations of 500 ng/mL (A) and 10 ng/mL, respectively. The CV% values were mostly less than 15%. The largest dispersions were found for the t-CIN measurements in oral fluid samples obtained via drooling (between 15.4 and 19.6%) and for CUS when the commercial Quantisal^®^ device (Abbot Toxicology, Chicago, IL, USA) was used (26.7%). Finally, in this section, the normalization of the areas in the calculation of the ME seems to partially correct it.

### 2.2. Experimental Part 2: Recovery of Alkaloids from Quantisal^®^ Pad

At concentrations between 10 and 100 ng/mL, EME and CUS were not detected either when the oral fluid was spiked on the pad (Tube a) and directly in the Quantisal^®^ buffer (Tube b) (Table 3). At 500 ng/mL, EME and CUS were recovered at 90% and above; the CV% for CUS was 52.2%. The remaining coca leaf alkaloids, namely TRO, COC, and t-CIN, at concentrations between 10 ng/mL and 100 ng/mL, showed extraction percentages ranging from 45% to 62% when the recovery was calculated with the absolute analyte areas. These percentages raised values between 76% and 112% when the chromatographic areas of the analytes were normalized using the deuterated IS (COC-d3). The results obtained for EME using normalized or absolute areas were not significantly different at 500 ng/mL.

**Figure 2:**
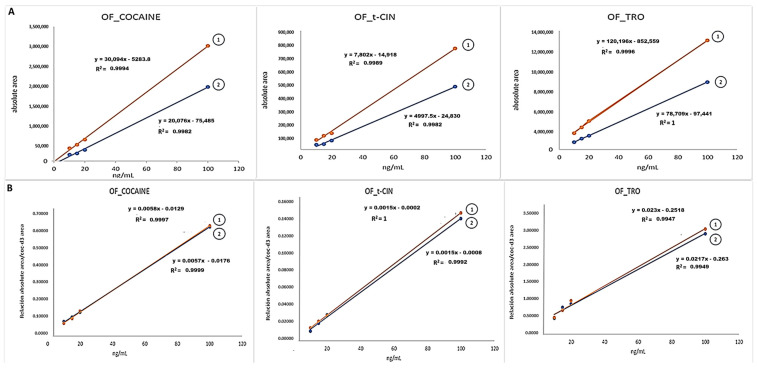
(**A**) Absolute area vs. concentration. Tube b: area buffer (with OF spiked with analytes); Tube a: area (pad (OF spiked with analytes) + buffer). (**B**) Normalized area ratio vs. concentration. Tube b: area buffer (OF spiked with analytes)/area COC-d3); Tube a: area (Pad (OF spiked with analytes) + buffer)/area COC-d3.

Complementarily to this experiment, Figure 2A shows the relationship between the absolute area versus concentration [spiking experiments on the pad (Tube a) and on the Quantisal^®^ buffer (Tube b)], whereas Figure 2B shows the results when using the normalized area ratio versus concentration. The concentration range for all analytes was from 10 ng/mL to 100 ng/mL. The calibration slopes for the experiments absent of IS (Figure 2A) were higher for the spiked buffer solutions than those obtained when spiking the pad. However, as shown in Figure 2B, similar calibration slopes were observed for the experiments involving a spiked buffer and pad (Tubes a and b) when normalized areas were used for assessing the analytical recovery.

### 2.3. Experimental Part 3: Process Efficiency or Apparent Recovery (R_A_) and Extraction Recovery (R_E_) or Extraction Efficiency of Alkaloids from Oral Fluid and Quantisal^®^

**Figure 3:**
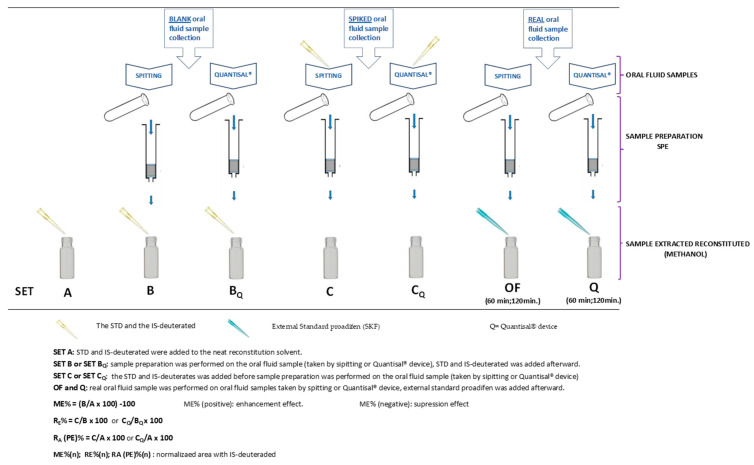
Graphical representation of the different sets performed for calculation matrix effect, recovery (RE), process efficiency (RA or PE), and real samples.

Table 4 and Table 5 show the results regarding the parameter process efficiency or apparent recovery (R_A_ %). The equation for this can be found at the bottom of Table 4 and Table 5 and the graphical description in Figure 3. Process efficiency (R_A_ %) is not typically measured in validation methods. First, the percentage values of process efficiency (R_A_ %) are higher for when the oral fluid samples were taken via drooling than using the Quantisal^®^ device. Secondly, the R_A_ % percentages go up when the analyte area is normalized with IS-deuterates (Table 4 and Table 5). The use of deuterated IS, which is not an isotopically labeled version of the molecule in the context of TRO (COC-d3 or EME-d3), had no influence on the results (Table 4), possibly because the R_A_% values for COC-d3 and EME-d3 are quite similar. Third, some R_A_ % and R_A_ (n)% have values >100%, specifically more than the 115% obtained in CUS′ R_A_ (n) (Table 4): 141%; t-CIN′ R_A_ % (126.5% (Table 4) and 190% (Table 5)) and t-CIN′ RA%(n) 138.2%; 157.8% (Table 4) and 127%; 159% (Table 5). Finally, no results were obtained when using the Quantisal^®^ device at concentrations between 10 ng/mL and 100 ng/mL (Table 5).

Table 6 and Table 7 show data about the extraction recovery, extraction efficiency, and true recovery (R_E_%) for the two oral fluid sampling procedures: drooling (Table 6) and the use of the Quantisal^®^ device (Table 7). Detailed recovery calculations (R_E_) can be found at the end of Table 4. Better recoveries were obtained when samples were collected by passive drooling and direct analysis than when oral fluid samples were extracted after Quantisal^®^ device collection. As shown in Table 7, EME, EME-d3, and CUS were not detected at concentrations of 10, 20, and 100 ng/mL when using the Quantisal^®^ device, and the recoveries were low at 500 ng/mL (9.6%, 19.6%, and 33.5% for EME, EME-d3, and CUS, respectively).

The recoveries (R_E(n)_%) calculated considering the normalized area (use of IS) were markedly higher than the recoveries (R_E_%) calculated with the absolute areas (Table 6 and Table 7).

### 2.4. Experimental Part 4: Limit of Detection and Limit of Quantification (LOD and LOQ)

Table 8 shows the limit of detection (LOD) and limit of quantification (LOQ) values obtained for EME, CUS, TRO, COC, and t-CIN. The nominal concentrations used for spiking the oral fluid samples were 2.5, 5, 10, 20, 50, 100, and 500 ng/mL. For both oral fluid sample collection methods (drooling and Quantisal^®^, the latter after analyte spiking onto the pad), a further SPE stage was performed.

### 2.5. Experimental Part 5: Application to Real Samples

**Figure 4:**
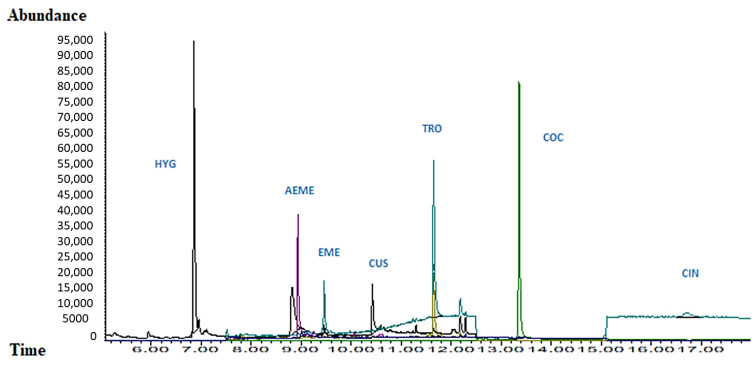
Chromatogram (GC-EI/MS). Coca leaf alkaloids in coca tea extract.

To demonstrate the applicability of the method, real samples were collected from two volunteers who drank a cup of coca tea Simultaneous samples of oral fluid (collected by drooling and using the Quantisal^®^ device) were taken at 60 and 120 min after the ingestion the cup of coca tea. HYG, CUS, and t-CIN were not detectable after 120 min when Quantisal^®^ was used, but they remained in the samples obtained by drooling. TRO was negative in all samples, while COC always remained detectable, although with the use of Quantisal^®^, the relationship was less strong (Table 9). At the time of these experiments, no certified commercial HYG control was available; thus, a coca leaf extract was used for identification (Figure 4).

## 3. Discussion

### 3.1. Experimental Part 1: Matrix Effect (ME)

**Figure 5:**
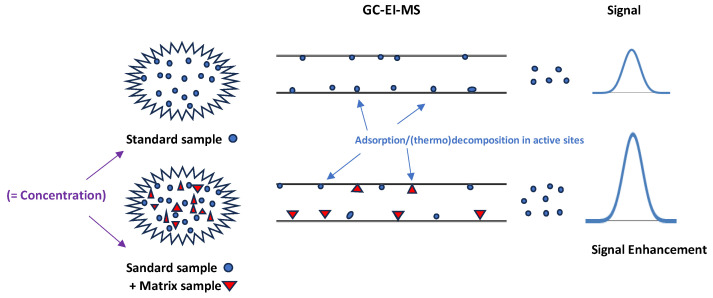
Schematic illustration of the matrix effect (determined via GC-EI-MS).

The analytical feature of the ME is a validation parameter included in several international guides, such as GTFC [14] and SWGTOX [15], when liquid chromatography–tandem mass spectrometry (LC-MS/MS) with electrospray ionization (ESI) is used as an analytical technique. However, the ME is not commonly assessed when using GC-MS techniques, since the ME in GC-MS usually leads to the enhancement of the analytical response [16]. Figure 5 shows a graphical representation of this phenomenon.

For the calculation of the ME%, we used the equation proposed by Matuszewski BK. et al. [17], with slight modifications: ME% = [(B/A − 1)] × 100. Our results are expressed in absolute ME%; positive values imply signal enhancement, while a negative ME% corresponds to signal suppression.

The limited knowledge about the ME when assessing HYG, CUS, CIN, and TRO by GC-MS led to the inclusion of the ME as one of the validation parameters, due to the fact that the ME has a high impact on the accuracy and the sensitivity of an analytical method. We did not find reference values for the ME in the reported guidelines for GC-MS [14,15]. Therefore, we have compared our results with the ME reference values for LC-MS/MS, and we have considered that the ME takes place when the average signal suppression or enhancement is higher than +/−25%, or when the CV% of the suppression or enhancement exceeds 15% (20% near the limit of quantification) [15]. Other authors have reasoned that MEs about +/−20% are close to repeatability values, and consequently, they can be considered as a lack of an effect [18].

Table 1 and Table 2 show that the analytes EME, TRO, and COC do not exceed the limits of the guidelines for the ME (LC-MS/MS) when the oral fluid samples are collected by drooling or the use of the Quantisal^®^ device, whereas the ME (enhancement) was observed for CUS and t-CIN. The ME enhancement is quite variable for CUS (Table 1: 21% to 576%; Table 2: 35% to 130%), and a relationship with the CUS concentration (10, 20, 50, 500, 1000, and 2000 ng/mL), even when measuring was performed at the same concentration (20 ng/mL: 38–576% and 500 ng/mL: 19–68%), could not be established. In addition, there is no correlation with the oral fluid collection procedure. Regarding t-CIN, the ME (analytical response enhancement) was also found (Table 1: 3% to 80%; Table 2: 18% to 46%), and the magnitude of the enhancement was independent of the t-CIN concentration and sample collection.

The use of a stable isotope-labeled internal standard (deuterated IS) was not enough to compensate the ME enhancement. The ME percentages were far from 0% in almost all CUS and t-CIN concentrations because the deuterated IS used to normalize the areas was different from the analyte (CUS/EME-d3 and t-CIN/COC-d3). However, the use of deuterated IS was found to be adequate for signal compensation (ME enhancement) when assessing in COC and EME since the same deuterated analogue was used for normalization.

The ME enhancement in GC-MS is explained by the adsorption and/or degradation of analytes on the active sites of the instrument: the inlet liner, column, and/or EI ion source. The interaction between targets (analytes) and instrument’s pieces is lower when the sample matrix is present. Matrix components compete with the analytes for the instrument’s active sites, and therefore, a higher amount of analyte reaches the detector (Figure 5) [19,20].

The poor precision of the data obtained regarding the ME% values for CUS and t-CIN (Table 1 and Table 2) is consistent with the concept of the phenomenon of “matrix-induced chromatographic response enhancement” in GCMS methods, where the active sites (in the liner and column) that are responsible for this effect are largely unpredictable between injections [20].

Further studies are needed to elucidate the adsorption and/or CUS and t-CIN degradation mechanisms in GC-MS that affect the analytical responses of CUS and t-CIN in oral fluid samples (drooling and Quantisal^®^ device) and the way to neutralize this effect.

### 3.2. Experimental Part 2: Recovery of Alkaloids from Quantisal^®^ Pad

In this experiment, we studied the percentages of analytes that were transferred from the Quantisal^®^ pad (spiking experiments with oral fluid and analytes) to the buffer by comparing the absolute signal (area) of the analytes extracted from Tube a (buffer + pad) versus the absolute signal (area) of Tube b (buffer spiked with the analytes).

Table 3 shows that when the Quantisal^®^ device is used within a concentration range spanning from 10 ng/mL to 100 ng/mL, neither EME nor CUS was detected in Tube a or Tube b. However, if we compare these results with those listed in Table 8 (limits of detection and quantification), which pertain to analyzing oral fluid collected by drooling, we can conclude that both CUS and EME could be partly adsorbed on the pad (Tube a) in the 10–100 ng/mL range. In addition, we can also speculate that there are some interferences from the buffer because CUS and EME were not detected in the buffer either (Tube b). The CUS and EME concentrations at 500 ng/mL showed recoveries of 90%. Concentrations at 500 ng/mL and higher are typically found in oral fluid contaminated with coca leaves or coca tea [4,21,22]. TRO, COC, and t-CIN showed recoveries from the pad close to 50% when the absolute areas were used, while recoveries higher than 90% were obtained using the IS approach (R(n) in Table 3. Figure 2 shows the effect of using absolute or normalized areas when assessing target recovery. Figure 2A shows lower calibration slopes for the spiking experiments with the pad (Tube a) than for the spiking experiments with the buffer (Tube b) for TRO, COC, and t-CIN, which implies lower analyte recovery from the pad (Tube a) than from the buffer (Tube b). However, there were no differences in the calibration slopes (Tube a and b) when normalizing using the deuterated IS (Figure 2B). The use of an IS-deuterated analogue with respect to the target improves the compensation of the response, and the calibration graphs for COC (Tube a and b) have an almost perfect overlap, while TRO and t-CIN have a slight difference in slopes since the IS-deuterated analogue used was COC-d3.

According to the results obtained, the use of Quantisal^®^ as a device for oral fluid sampling and the sampling of further coca leaf alkaloids would not be suitable due to its low sensitivity when detecting CUS, which is one of the markers proposed to differentiate legal coca leaf consumption versus the illicit consumption of cocaine.

### 3.3. Experimental Part 3: Process Efficiency or Apparent Recovery (R_A_) and Extraction Recovery (R_E_) or Extraction Efficiency of Alkaloids from Oral Fluid and Quantisal^®^

The analytical parameter recovery (R_E_) and process efficiency (R_A_) defined by Matuszewski and co-workers [17] were assessed to test the method’s performance. Some clarifications about the way one can calculate the recovery are needed, since these parameters can be erroneously calculated and create misinterpretation when comparing results among authors.

Firstly, these parameters present some confusion in the literature because recovery, also called extraction recovery (R_E_) (extraction efficiency or true recovery), is calculated as process efficiency (R_A_) by some authors [18]. The R_E_ is calculated as C/A instead of C/B (Figure 3), and the error due to the matrix effect is not considered when the C/A × 100 ratio, instead of the C/B × 100 ratio, is used in the R_E_ calculation. R_E_ values reported in the literature that are higher than 100% are a clear indication that the matrix effect enhancement was not considered for R_E_ assessment [17,18].

A quick comparison between the data listed in Table 4 and Table 5 (R_A_ = process efficiency) and Table 6 and Table 7 (R_E_ = extraction recovery) show that higher R_A_ values (Table 4 and Table 5) than those listed in Table 6 and Table 7 for R_E_ are obtained at the same concentration, which demonstrates that matrix effect contributions are not considered for calculations based on R_E_. The most noticeable difference between R_A_ and R_E_ was found for t-CIN and CUS due to an enhanced matrix effect for these two targets, as reported in “Experimental part 1: matrix effect”. Table 4 and Table 5 (experiments for passive drooling and Quantisal^®^ sampling, respectively) show R_A_ values higher than 100%: 126.5% (Table 4) at 50 ng/mL and 190% (Table 5) at 500 ng/mL. These values contrast with the R_E_ ratios (Table 6 and Table 7) for t-CIN at 50 ng/mL (26.2%, Table 6) and at 500 ng/mL (129%, Table 7). On the other hand, CUS shows R_A_ values between 60% and 104% at 500 ng/mL for oral fluid sample collection by drooling (Table 4) and R_E_ values of 36% for the same concentration and sampling method (Table 6).

The smallest differences between R_A_ and R_E_ were found for analytes showing a low or no matrix effect, such as EME, TRO, and COC, as was reported in “Experimental part 1: matrix effect”. This was the expected result according to the Matuszewski et al. equation for process efficiency, (PE) (R_A_) (%) = C/A × 100 = (ME × RE)/100 [17].

**Figure 6:**
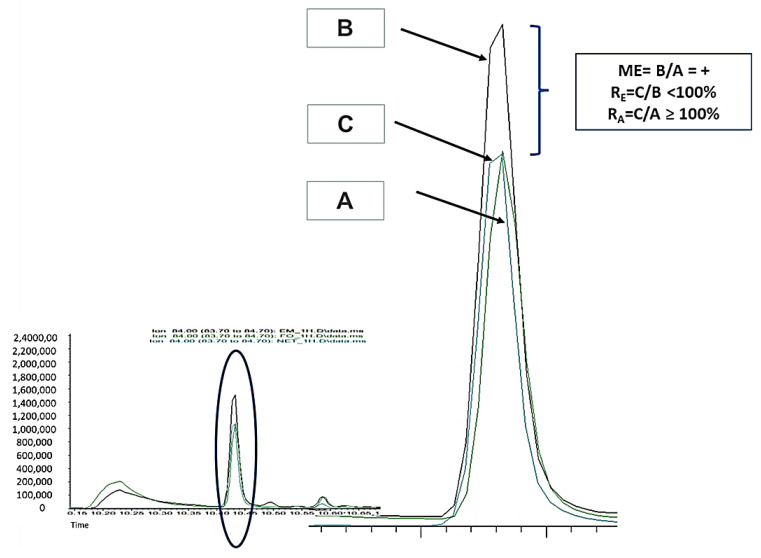
Chromatogram GC-EI/MS. (**A**) Cuscohygrine standard 500 ng/mL. (**B**) Oral fluid sample spiked with cuscohygrine 500 ng/mL after extraction. (**C**) Oral fluid sample spiked with cuscohygrine 500 ng/mL before extraction.

Another source of confusion is the use of normalized analyte areas (use of an internal standard) instead of absolute areas in R_E_ calculation, as is highly recommended by some guidelines, such as the GTFCh and FDA Guidelines [14,23]. The normalized area compensates for the analyte response to the internal standard response and can lead to “artificially” high recoveries that are not true from an analytical point of view. This effect can be visualized in Figure 6 for CUS for the spiking experiments before (peak C) and after (peak B) extraction.

The results in Table 6 and Table 7 show the effect on the increase in recovery when the areas are normalized with an internal standard (deuterated IS, (R_E_ %(n)). Calculating several R_E_ % values using absolute areas leads to R_E_ %(n) values that are between two and five times higher than those obtained when using normalized areas: 46% vs. 119% for COC at 50 ng/mL (Table 6), 9.6% vs. 57.3% for EME at 500 ng/mL (Table 7), and 53% vs. 92% for CUS at 50 ng/mL (Table 6).

Specifically, if we analyze the COC results for Quantisal^®^ device sampling at the tested concentrations of 10 ng/mL, 20 ng/mL, and 100 ng/mL (Table 7), the calculated R_E_ % values in accordance with international guidelines are between 25% and 40%, but the recoveries improve “artificially” between 94% and 104% when normalizing the areas (Table 6 and Table 7). As an example, the reported R_E_ % values for COC in oral fluid (Quantisal^®^ device sampling) calculated by using normalized values were 95.7% [8] and 97% [24], values which are in good agreement with our results (Table 7).

Similar results were obtained for other targets (Table 4, Table 5, Table 6 and Table 7), and improved R_A_ values were obtained when using normalized areas (deuterated IS): the R_A_ (n) values were above 100% (113.8% to 157.8%) at concentrations between 5 ng/mL and 500 ng/mL for t-CIN (drooling sampling), and R_A_ values of 127% and 159% were achieved at concentrations of 10 ng/mL and 500 ng/mL, respectively, when the Quantisal^®^ device are used.

The best performance in terms of the R_A_ and R_E_ results (shown in Table 4, Table 5, Table 6 and Table 7) was obtained with oral fluid sampled by drooling. Neither EME nor CUS (10, 20, and 100 ng/mL) were detected when the Quantisal^®^ device was used (Table 5 and Table 7). These results are in good agreement with those listed in Table 2 and Table 5.

Comparisons regarding recovery (R_E_ or R_A_ for CUS, TRO, and t-CIN (Quantisal^®^ device)) in oral fluid cannot be established, since our results (Table 4, Table 5, Table 6 and Table 7) are the first ones to be reported in the literature in this context. Similarly, there were not references regarding EME (a COC metabolite and coca leaf alkaloid).

It must be mentioned that the analytical parameters for the recovery (R_E_) and the efficiency of the process (R_A_) are not included in many method validation guidelines, and there are no references for establishing a clear criterion for acceptance/rejection. In addition, R_A_ is not commonly used as a validation performance parameter [18]. FDA, ICH, and M10 guidelines, as well as other authors [23,25,26], state that “recovery of the analyte does not need to be 100%, but the extent of recovery of an analyte and the IS should be consistent”, whereas the GTFCh guideline [14] establishes that R_E_ should be high and preferably over 50%.

Our main conclusion for this section is that the Quantisal^®^ device is not suitable for the purpose of this work since EME and CUS are not detectable at low concentrations (an expected scenario for individuals chewing coca leaf or drinking coca tea). EME and CUS have problems with respect to desorption from the pad, and interferences from the Quantisal^®^ buffer cannot be ruled out either. An explanation for this can be given by the polarity of EME and CUS, which are more polar than COC, TRO, and t-CIN, as measured by their Log P values. Log P or K_ow_ describes the polar or hydrophobic character of a compound according to its octanol/water distribution (hydrophobicity increases with higher values of Log P, and compounds or solvents with low or negative Log P indicate polarity). The Log P values for EME and CUS are −0.23 and 0.72, respectively, whereas COC, t-CIN, and TRO show high Log P values (2.6, 2.7, and 2.6, respectively), which implies higher hydrophobicity.

Finally, low precision and repeatability (CV% values higher than 15% in Table 6 and Table 7) was obtained for CUS when assessing the extraction recovery (R_E_%). The remaining analytes showed CV% values for R_E_% (Table 6 and Table 7) lower than 15%. We have taken the upper acceptable limit of CV% arbitrarily to be 15 or 20% (near the detection limit).

### 3.4. Experimental Part 4: Limit of Detection and Limit of Quantification (LOD and LOQ)

**Figure 7:**
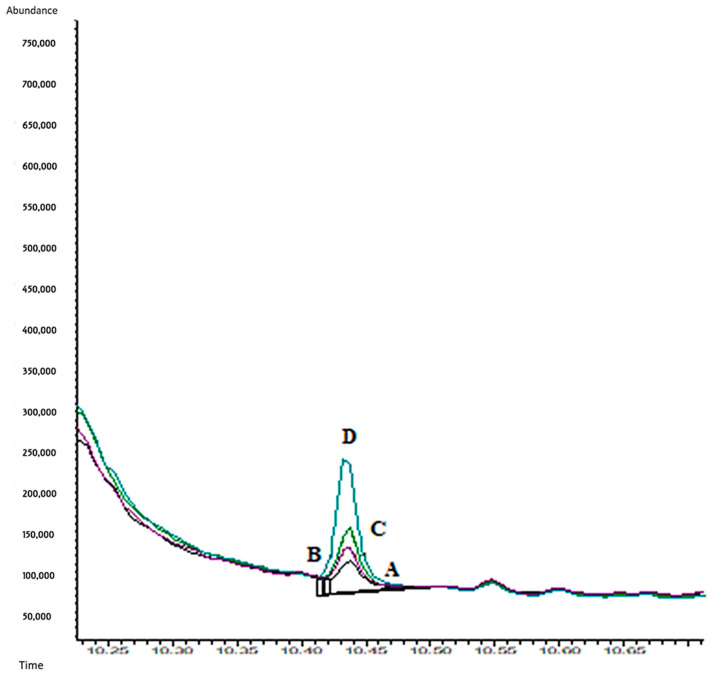
Chromatogram (GC-EI/MS). Cuscohygrine (OF sample). A. 2.5 ng/mL; B. 5 ng/mL; C. 10 ng/mL; D. 20 ng/mL (monitorized ion *m*/*z* 84).

Table 8 shows similar detection and quantification limits for TRO and COC when oral fluid samples were collected by drooling and by using the Quantisal^®^ device (LOD of 5 ng/mL and LOQ of 10 ng/mL). However, t-CIN showed better performance after drooling collection (LOD of 5 ng/mL and LOQ of 10 ng/mL vs. and LOD of 10 ng/mL and a LOQ of 20 ng/mL for Quantisal^®^ device sampling). On the other hand, EME and CUS showed the worst results when the Quantisal^®^ device is used. Although we need more concentration levels between 100 and 500 ng/mL for an accurate calculation of LOD and LOQ with the Quantisal^®^ device, obviously, they will be several times higher than the LOD and LOQ values for drooling oral fluid sampling, where the best performance for EME and CUS was obtained. Figure 7 shows a chromatogram (CG-EI-MS) for the oral fluid’s samples spiked at different CUS concentrations.

We can speculate that the poor results obtained with the use of the Quantisal^®^ device are due to the fact that EME and CUS are more polar alkaloids than TRO, COC, and t-CIN, and they were more likely to be retained on the Quantisal^®^ pad (moreover possible interferences from the Quantisal^®^ buffer). The recovery results shown in Table 6 for EME and CUS are in good agreement with these findings. The use of more concentration levels between 100 and 500 ng/mL was not considered since the expected analyte concentration in oral fluid taken from people who chew coca leaf or drink coca tea is generally lower than 100 ng/mL [22].

As previously commented, the findings for COC after Quantisal^®^ device sampling are in good agreement with those reported in the literature [8,9]. We found no information regarding other analytes such as TRO, t-CIN, EME, and CUS in oral fluid subjected to GC-MS analysis, and this was also true for when the samples were collected by drooling or the use of the Quantisal^®^ device. Our findings show improved LOD and LOQ values for COC, TRO, t-CIN, EME, and CUS pertaining to when oral fluid samples were collected by drooling, followed by SPE (Oasis^®^ HLB extraction cartridge) and liquid chromatography–tandem mass spectrometry (LC-MS/MS) analysis [22].

### 3.5. Experimental Part 5: Application to Real Samples

The aim of this study was to evaluate two oral fluid sampling procedures (drooling and the use of a Quantisal^®^ device) coupled with SPE for clean-up purposes and GC-MS analysis. One of the goals for accepting the developed method as reliable for establishing the consumption of coca leaves via chewing and via coca tea drinking is that CUS and HYG can be detectable within the same time window wherein COC is detectable, which implies that HYG and CUS should not be lower than the LOD before the COC concentrations were below the COC cut-off values established by international guidelines for oral fluid (8 ng/mL or lower in DUID and WDT) [11,12]. Our real oral fluid sample analysis results are in good agreement with those previously shown. The use of the Quantisal^®^ device used in this study for oral fluid sample collection does not meet the established requirements, since HYG, CUS, t-CIN, and EME were not detectable in oral fluid samples collected at 120 min after coca leaf consumption in volunteer 1, whereas COC remained positive. Regarding TRO, undetectable concentrations were also found, which matches with the low TRO concentrations in coca leaves (Table 9).

The drooling sample collection technique seems to meet the necessary requirements for method validation. However, some important issues should be considered for accepting the developed method as a reliable procedure for distinguishing between coca leaf (chewing) and coca tea consumption and cocaine abuse. Firstly, the testing time should be longer to verify whether CUS and HYG are positive while COC is still detectable. Secondly, international guidelines on workplace drug testing (WDT) and driving under the influence of drugs (DUID) programs offer cut-off values for COC and benzoylecgonine (BEC), which is one of the main COC metabolites and was not measured in our work because a derivatization stage is necessary for BEC determination by GC-MS. Although some of the limitations of the developed method could be overcome by using LC-MS (and LC-MS/MS), most forensic laboratories worldwide, and mainly those in Latin American countries, use GC-MS instrumentation for drugs analysis. Therefore, our efforts were focused on solving some limitations of GC-MS when assessing coca alkaloids in oral fluid.

The analysis of oral fluid samples allows us to confirm that a person has consumed coca leaves through the presence of the markers HYG and CUS, as well as t-CIN as a secondary marker, but we are not able to rule out whether an individual has consumed cocaine illegally at the same time.

## 4. Materials and Methods

### 4.1. Reagents and Standards

COC, EME, t-CIN, and TRO (1 mg/mL in acetonitrile), cocaine-d3 (COC-d3), and ecgonine- methyl ester-d3 (EME-d3) (0.1 mg/mL in acetonitrile) were obtained from LGC Standards, S.L.U. (Barcelona, Spain). CUS (10 mg) was obtained from Toronto Research Chemicals Inc. (North York, ON, Canada). HYG was not commercially available at the time of this study. It was identified based on *m*/*z* (precursor ion) → *m*/*z* (product ion) transitions and obtained after the infusion of a coca leaf extract and mass spectral recording. Methanol (LC-MS grade) was purchased from Riedel-de Haën (Seelze, Germany), ammonium formiate was purchased from Fluka Analytical (Steinheim, Germany), and acetic acid was purchased form Merck (Madrid, Spain). Boric acid, potassium chloride, sodium hydroxide, and the reagents necessary for the preparation of the borate buffer were also supplied by Merck. Ultrapure water of 18 MΩcm resistivity was obtained from a water purification device from Millipore Co. (Bedford, MA, USA). A WATERS OASIS^®^ HLB extraction cartridge of 3 cc/60 mg came from Waters, Spain. The Quantisal^®^ saliva collection device was supplied by Immunalysis Corporation (Pomona, CA, USA). Proadifen (SKF) was used as an external standard, and it was supplied by Merck (Madrid, Spain).

### 4.2. Working Solutions

Three working standard calibrations were prepared in acetonitrile from stock solutions at concentrations of 10, 1, and 0.1 µg/mL for COC, EME, t-CIN, TRO, and CUS, respectively. A mix of deuterated internal standard (COC-d3, EME-d3) was prepared in acetonitrile from stock material at a concentration of 1.5 µg/mL. All working standards were stored at −20 °C when not in use. The mix of deuterated standards were added at a final concentration of 15 ng/mL. SKF (external standard) was prepared at a concentration of 1 µg/mL.

### 4.3. Instrumental Analysis (GC-EI/MS)

Analysis was performed using a gas chromatography model 7890B from Agilent Technologies (Las Rozas, Spain) combined with an Agilent 5977B mass spectrometer. The ionization source employed was electron impact ionization. An HP5-MS capillary column (30 m × 250 µm i.d., 0.5 µm film thickness; Agilent Technologies) with helium as the carrier gas (1 mL/min) was used for separation via gas chromatography. The interface, source, and quadrupole temperatures were 300 °C, 250 °C, and 150 °C, respectively. The fixed electron energy was 70 eV, and the injector temperature was 250 °C. The temperature program was as follows: the temperature was maintained at 70 °C for 3 min; the temperature was increased to 270 °C at 25 °C/min and was held to 270 °C for 7 min. The injection volume was 1 µL. Samples were injected in splitless mode. All compounds were injected in full SCAN mode for identification (mass spectra and retention time), and then the analysis was performed in SIM (selected ion monitoring) mode to increase the sensibility of the method. The ions selected from their main fragmentation pattern according to their abundance are shown in Table 10.

### 4.4. Sample Collection

Ten drug-free oral fluid samples were collected from laboratory volunteers and were used for method development according to international criteria [14,15].

Real oral fluid samples were also collected from 2 volunteers after they drank a cup of coca tea. The coca tea used by all volunteers was from the same brand and batch. Informed consent was obtained from all the volunteers.

### 4.5. Sample Preparation

The oral fluid samples collected by the spitting technique (drooling) were centrifugated at 10,000 rpm for 10 min. On the other hand, the oral fluid samples collected using the Quantisal^®^ device were processed used three different procedures:

a. One mL of oral fluid sample was spiked on the Quantisal^®^ device pad at different concentrations of CUS, EME, TRO, COC, t-CIN, and the internal standard (IS: mix of COC-d3 and EME-d3; 10 µL Sol. 1.5 µg/mL). The protocol we followed adhered to the manufacturer’s specifications and was as described by Cohier et al. [24], except for the fact that we used 1 mL of buffer instead of 3 mL. In addition, blank OF samples were processed in the same way.
b. One mL of oral fluid sample containing different concentrations of CUS, EME, TRO, COC, t-CIN, and IS (mix of COC-d3 and EME-d3; 10 µL Sol. 1.5 µg/mL) was added directly to one mL of Quantisal^®^ preservative buffer (without pad) over 24 h at 4 °C. In addition, blank oral fluid samples were processed in the same way.
c. One mL of oral fluid sample was taken from volunteers who had previously drunk coca tea using the Quantisal^®^ device. Then, the sample was transferred to a 10 mL glass tube and mixed with 10 µL of 1.5 µg/mL internal standard (IS) (mix of COC-d3 and EME-d3).

Then, 1 mL of borate buffer pH 9.2 was added to each sample obtained after applying the different procedures (a, b, and c). After shaking the mixture, the extraction process was carried out by solid-phase extraction (SPE).

In addition, as a control, a mixture of coca leaves was prepared. The coca leaves were cut into small pieces (50 mg) and mixed with 5 mL of methanol/acetonitrile/2 mM ammonium formate (25:25:50, *v*/*v*/*v*). The mixture was mechanically stirred for 15 min. Subsequently, the liquid phase was loaded into a SPE cartridge. The evaporated extract was diluted with methanol. This extract was mainly used as a control for the alkaloid HYG and as additional verification for the alkaloids COC, t-CIN, EME, CUS, and TRO.

### 4.6. Extraction via SPE

Each SPE cartridge (Oasis^®^ HLB Extraction Cartridge, 3 cc, 60 mg; Waters) was conditioned with 2 mL of methanol and 2 mL of distilled water. Then, 1 mL of oral fluid mixed with IS and borate buffer pH 9.2 was loaded into the cartridge. Washing was performed using 2 mL of a mix of 5% methanol in distilled water. The cartridges were then dried under vacuum for 10 min. The studied compounds were eluted with 2 mL of methanol followed by 2 mL of 2% acetic acid in methanol. The eluent was evaporated at 40 °C under a stream of nitrogen gas. The dried extract was reconstituted in a final volume of 50 µL with methanol. The reconstituted samples were transferred to autosampler vials for GC-MS injection.

### 4.7. Experimental Study

The experimental study was divided into 5 different parts to study the Matrix Effect (ME), recovery, process efficiency, limits of detection and quantification, and the application to real samples.

#### 4.7.1. Experimental Part 1: Matrix Effect (ME)

Two different sets (A and B/B_Q_) of oral fluid samples were prepared (Figure 3).

Set A: Neat standard solutions were prepared at different concentrations (10, 20, 50, 500, 1000, and 2000 ng/mL for CUS; 10, 20, 50, and 500 ng/mL for EME, TRO, COC, and t-CIN). Deuterated IS was added to all neat standard solutions at a final concentration of 15 ng/mL. This standard was injected into the GC-MS device three times to establish a mean peak area for each concentration.

Set B: Blank oral fluid samples were extracted via SPE. Once the process extraction was complete, each extract was spiked in triplicate (n = 3) or sextuplicate (n = 6) on two different days (20, 500, 1000, and 2000 ng/mL for CUS; 20 and 500 ng/mL for TRO, EME, COC, and t-CIN). Deuterated IS was added to all extracts at a final concentration of 15 ng/mL. Blank OF samples were also analyzed. The final volume of the fortified matrix samples was 50 µL of methanol, and 1 µL was injected into the GC-MS device.

Set B_Q_: One mL of blank oral fluid was added to the Quantisal^®^ device according to sample preparation (a). Once the extraction process was complete, each matrix sample was fortified in triplicate (n = 3) at concentrations of 10, 20, and 50 ng/mL for CUS, TRO, EME, COC, and t-CIN. Deuterated IS was added to all matrix sample extracts at a final concentration of 15 ng/mL. The final volume of the fortified matrix samples was 50 µL of methanol, and 1 µL was injected into the GC-MS device.

The equations used for the ME calculations are shown in Table S1 of the Supplementary Materials. A negative result indicated suppression, and a positive result indicated an increase in the analyte signal.

#### 4.7.2. Experimental Part 2: Recovery of Alkaloids from the Quantisal^®^ Device (Using Pad)

The oral fluid pool was centrifuged at 10,000 rpm, and the supernatant was collected. Different concentrations of the analytes under study were added to the aliquots taken from the supernatant (CUS, EME, COC, TRO, and t-CIN at concentrations of 0 (blank), 10, 15, 20, 100, and 500 ng/mL). Deuterated IS (mix of COC-d3 and EME-d3; 15 ng/mL) was also added. One milliliter of each aliquot was added to the Quantisal^®^ device according to the design of the previous procedures (sample preparation a and b). The samples were refrigerated at +4 °C for 24 h. The samples were then extracted by SPE. The extracts were reconstituted with 50 µL of methanol, and 1 µL was injected into the GC-MS device. The samples were analyzed in triplicate.

The equations used for the recovery calculations are shown in Table S2 of the Supplementary Materials. It was expressed as a percentage for each compound.

#### 4.7.3. Experimental Part 3: Process Efficiency or Apparent Recovery (R_A_) and Extraction Recovery (R_E_) or Extraction Efficiency of Alkaloids from Oral Fluid and the Quantisal^®^ Device

Triplicate oral fluid samples were spiked before (set C) and after SPE (set B) at concentrations of 0, 5, 10, 20, 50, and 500 ng/mL for CUS, EME, COC, TRO, and t-CIN. Deuterated IS (a mix of COC-d3 and EME-d3) was also added at a concentration of 15 ng/mL. Triplicate samples of standard solutions (set A) at 5, 10, 20, 50, and 500 ng/mL with deuterated IS were also analyzed (Figure 3).

On the other hand, 1 mL of oral fluid was fortified at 10, 20, 50, and 500 ng/mL and added to the Quantisal^®^ pad until the blue indicator appeared in the window of the polypropylene stem. The pad was immersed in 1 mL of buffer, and the cap was tightened firmly to close the tube. The tube was shaken to ensure that the entire pad was soaked in the buffer. Samples were then extracted (Set C_Q_). The same procedure was carried out by fortifying the sample after extraction with Quantisal^®^ (Set B_Q_) at concentrations of 10, 20, 100, and 500 ng/mL.

The final volume of the fortified matrix samples was 50 µL of methanol, and 1 µL was injected into the GC-MS device. Blank oral fluid samples were also prepared.

The recovery was expressed as a percentage for each compound. The recovery values were determined as shown in Table S3 in the Supplementary Materials.

#### 4.7.4. Experimental Part 4: Determination of Limit of Detection and Limit of Quantification (LOD and LOQ)

In triplicate, the oral fluid samples were spiked at concentrations of 0, 2.5, 5, 10, 20, 50, 100, and 200 ng/mL for EME, CUS, TRO, COC, and t-CIN. On the one hand, 1 mL aliquots were taken and subjected to SPE. On the other hand, another 1 mL of spiked oral fluid was introduced into the Quantisal^®^ Pad, placed in 1 mL of buffer, and extracted by SPE. The final volume of the fortified matrix samples was 50 µL of methanol, and 1 µL was injected into the GC-MS device.

The limit of detection and limit of quantification were determined according to a signal-to-noise ratio of 3 and a signal-to-noise ratio of 10, respectively [27,28].

#### 4.7.5. Experimental Part 5: Application to Real Samples

Real oral fluid samples were collected from 2 volunteers after they drank a cup of coca tea at 60 min and 120 min. A blank oral fluid was taken 10 min before drinking the cup of tea. Oral fluid samples were collected by spitting into a tube (1 mL or more). In addition, a second oral fluid sample was collected by drooling at the same time using a Quantisal^®^ device and following the procedure described in the manufacturer’s specifications and by Cohier et al. [24]. Both samples were then subjected to the extraction procedure mentioned earlier in this work.

At the end of the extraction procedure, 10 µL of external standard proadifen (SKF) and 40 µL of methanol were added. One microliter was injected into the GC-MS device. SKF was used as an external standard to normalize the areas of analytes. It was added after the extraction of the samples and before injection in GC-MS. SKF was not affected by the sampling procedure, allowing for it to be differentiated from the deuterated internal standard added prior to sampling (Figure 3).

The results are presented as the relation to absolute area of each analyte over the area of external standard (SKF). HYG identification was performed using extracted coca leaves.

## 5. Conclusions

The study of several alkaloids presents in coca leaves, such as hygrine, cuscohygrine, and cinnamoylcocaine, has been proposed to try to distinguish legal cocaine consumption (chewing coca leaves or drinking coca tea) from illegal cocaine consumption. For the first time, parameters such as the Matrix Effect (ME), recovery, process efficiency, and limits of detection and quantification have been evaluated for these compounds in oral fluid after solid-phase extraction. In addition, the proposed methodology was applied to real cases.

The matrix effect is a phenomenon that is not usually described in GC-MS. This work has revealed that only CUS and t-CIN show an increasing effect on the ME, so it should be reduced or eliminated as it affects the calculation of the total concentration. The remaining coca leaf alkaloids analyzed (COC, TRO, and EME) did not show a significant ME (<25%, calculated according to LC-MS/MS as there are no requirements for GCMS). Regarding the recovery values (R_E_) (absolute areas), it is remarkable that the use of the Quantisal^®^ device did not allow for the recovery of the analytes EME, EME-d3, and CUS in the range of concentrations tested (5 ng/mL to 100 ng/mL). Therefore, this device was not satisfactory for the determination of EME and CUS in oral fluid, while the recoveries of TRO, COC, and t-CIN ranged from 18% to 40%. However, when working with normalized areas, the recoveries increased to between 69 and 104% in the range from 10 to 100 ng/mL. The use of oral fluid collected by passive drooling employing SPE as the extraction technique and GC-MS as the instrumental technique has proven valid for application in workplace testing and drug-impaired driving (DUID) programs in Latin American countries. However, sample collection is complicated and unpleasant for the donor and the sampler.

These preliminary results were derived from our evaluation of the behavior of some coca leaf alkaloids not commonly used in forensic toxicology laboratories using GC-MS. Further research is required to understand the certain limitations found in this work, such as the matrix effect and the greater dispersion found for CUS, which could be due to adsorption and/or degradation processes in the active sites of GC-EI/MS and/or to the extraction processes carried out in the laboratory, and further research should be carried out to understand if the polarity of CUS, EME, and EME-d3 makes their extraction difficult using Quantisal^®^ devices.

## Figures and Tables

**Table 1:** Matrix effect (ME%): Oral Fluid samples (1 mL) taken by passive drooling (spitting), extracted by SPE cartridge and after spiked by analytes and IS-d3.

	ME (%)	ME (n) (%)	ME (%)	ME (n) (%)	ME (%)	ME (n) (%)	ME (%)	ME (n) (%)	ME (%)	ME (n) (%)	ME (%)	ME (%)
Nominal Conc. (ng/mL)	EME	EME_n_ (EME_d3)	CUS	CUS_n_ (EME_d3)	TRO	TRO_n_ (EME_d3)	COC	COC_n_ (COC_d3)	t-CIN	t-CIN_n_ (COC_d3)	COC-d3	EME-d3
2000	-	-	21	-	-	-	-	-	-	-	-	-
CV% (n = 3)	-	-	5.8%	-	-	-	-	-	-	-	-	-
1000	-	-	64	-	-	-	-	-	-	-	-	-
CV% (n = 3)	-	-	13.0%	-	-	-	-	-	-	-	-	-
500	9; 21	−8; 14	19; 68	22; 36	13; 17	−7; 2	0–9	0	3; 47; 80	−5; −4; 21	9	21–34
CV% (n = 6)	2.5%	4.7%	12.7%	7.5	2.3%	3.4%	13.9%	1.4%	19.6%	17.9%	14.7%	10.3%
20	21; 26	15; −3	0	21; 385	4; 17	−5; −6	5–15	−1; 0	41; 31	21; 18	20	30
CV% (n = 6)	6.8%	2.3%	9.3%	10.6%	5.6%	7.8%	18.7%	12.6%	18.6%	15.4%	2.3%	4.9%

ME (%) = [(B/A − 1)] × 100; A: neat standard; B: Spiked sample after extraction from blank OF samples. ME (n) (%) = [(B/IS)/(A/IS) − 1] × 100; IS: internal standard; Subscript (n) meaning ME% IS deuterated normalized. Results > 0 indicates enhancement. Results < 0 indicates suppression.

**Table 2:** Matrix effect (ME%): Oral Fluid samples (1mL) taken with Quantisal^®^ and extracted by SPE cartridge and after spiked by analytes and IS-d3.

	ME_Q_ (%)	ME_Q (n)_ (%)	ME_Q_ (%)	ME_Q (n)_ (%)	ME_Q_ (%)	ME_Q (n)_ (%)	ME_Q_ (%)	ME_Q (n)_ (%)	ME_Q_ (%)	ME_Q (n)_ (%)	ME_Q_ (%)	ME_Q_ (%)
Nominal Conc. (ng/mL)	EME	EME_n_ (EME_d3)	CUS	CUS_n_ (EME_d3)	TRO	TRO_n_ (COC_d3)	COC	COC_n_ (COC_d3)	t-CIN	t-CIN_n_ (COC_d3)	COC-d3	EME-d3
10	13	nd	130	-	2	−9	16	3	46	30	12	nd
CV% (n = 3)	3.7%	nd	16.6%	-	2.1%	3.3%	4.3%	2.5%	6.4%	4.2%	5.3%	nd
20	2	nd	75	-	−4	−9	7	2	36	30	5	nd
CV% (n = 3)	5.5%	nd	6.8%	-	2.6%	1.5%	2.7%	0.5%	4.6%	2.2%	3.7%	nd
50	8	11	35	13	-	-	16	0	18	2	16	−3
CV% (n = 3)	6.0%	1.5%	26.7%	27.0%	-	-	3.1%	0.4%	2.6%	5.0%	2.7%	7.5%

ME_Q_ (%) = [(B_Q_/A − 1)] × 100; A: neat standard; B_Q_: Spiked sample after extraction from Quantisal^®^ oral fluid device. ME_Q_ (n) (%) = [(B_Q_/IS)/(A/IS) − 1] × 100; IS: internal standard; Subscript (n) meaning ME_Q_ (n) (%) IS deuterated normalized. Results > 0 indicates enhancement. Results < 0 indicates suppression.

**Table 3:** Recovery of coca leaf alkaloids from the Quantisal^®^ (OF sample device) Pad.

Nominal Conc.	R (EME)	Rn (EME/EME-d3)	R (CUS)	R (TRO)	Rn (TRO/COC-d3)	R (COC)	Rn (COC/COC-d3)	R (t-CIN)	Rn (t_CIN/COC-d3)	R (COC-d3)	R (EME-d3)
10 ng/mL	nd	nd	nd	45%	95%	52%	111%	51%	76%	47%	nd
15 ng/mL	nd	nd	nd	50%	112%	47%	107%	40%	91%	44%	nd
20ng/mL	nd	nd	nd	49%	90%	52%	96%	55%	102%	54%	nd
100 ng/mL	nd	nd	nd	62%	95%	64%	99%	62%	96%	64%	nd
500 ng/mL	93	91	90	69	54	77	93	83	101	83	110
CV% (n = 3)	11.6%	9.9%	52.2%	12.5%	10.8%	4.5%	3.9%	5.6%	2.9%	5.1%	8.9%

nd: not detectable; R(x): Relation area Tube a (Pad + buffer)/area Tube b (Buffer) × 100; (x) EME; CUS; TRO; COC; t-CIN; COC-d3; EME-d3; Rn (x/x-d3): Relation area (tube a/IS-d3)/area (tube b/IS-d3); (x): EME; CUS; TRO; COC; t-CIN; COC-d3; EME-d3; (x-d3): EME-d3, COC-d3.

**Table 4:** Process Efficiency or Apparent Recovery (R_A_) (%): Oral Fluid samples (1 mL) taken by passive drooling (spitting) and extracted by SPE cartridge. R_A_ (%) and R_A_ (n) (%).

	R_A_ (%)	R_A_ (n) (%)	R_A_ (%)	R_A_ (n) (%)	R_A_ (%)	R_A_ (n) (%)	R_A_ (n) (%)	R_A_ (%)	R_A_ (n) (%)	R_A_ (%)	R_A_ (n) (%)	R_A_ (%)	R_A_ (%)
Nominal Conc. (ng/mL)	EME	EME_n_ (EME_d3)	CUS	CUS_n_ (EME_d3)	TRO	TRO_n_ (EME_d3)	TRO_n_ (COC_d3)	COC	COC_n_ (COC_d3)	t-CIN	t-CIN_n_ (COC_d3)	COC-d3	EME-d3
5	67.5%	99.4%	nd	-	40.2%	58.7%	72.1%	61.5%	110.8%	65.8%	117.3%	55.4%	68.1%
CV% (n = 3)	9.3%	4.6%	-	-	11.6%	8.2%	8.0%	9.4%	2.4%	12.2%	6.7%	7.0%	7.4%
10	79%	94%	nd	-	48.9%	58.2%	65.7%	80.9%	111.1%	99.0%	138.2%	73.2%	83.9%
CV% (n = 3)	5.0%	6.2%	-	-	4.2%	2.7%	9.6%	10.9%	5.0%	25.3%	13.0%	13.0%	6.0%
20	53%	104%	nd	-	40.1%	78.6%	71.9%	47.4%	84.5%	64.2%	113.8	55.7	51.0%
CV% (n = 3)	5.6	2.8%	-	-	3.2%	4.3%	7.3%	12.7%	13.6%	22.4%	23.8%	4.8%	7.5%
50	70.6%	89.1%	73.0%	141.0%	45.5%	57.5%	55.1%	78.0%	95.6%	126.5%	157.8%	81.6%	79.2%
CV% (n = 3)	5.5%	1.5%	14.0%	6.2%	3.1%	3.2%	5.5%	9.5%	7.0%	15.6%	12.9%	5.8%	6.3%
500	41%	66%	60–104% (▪)	66–95% (▪)	-	-	-	-	-	-	-	-	-
CV% (n = 6)	6.9%	4.5%	16.8–22.3%	14.8–19.8%	-	-	-	-	-	-	-	-	-

RA (%) = (absolute area C/absolute area A) × 100; C: Spiked OF sample before extraction; A: neat standard. RA (n) (%) = (absolute area C/area IS)/(absolute area A/area IS) × 100; Subscript (n) meaning recuperations IS deuterated normalized. nd: not detectable; Percentages (%) results are the average of three OF samples spiked at each concentration and extracted by SPE and three neat standards injected. (▪) Percentages (%) results are the average of three OF samples spiked at each concentration and extracted by SPE and three neat standards injected and repeated in two days.

**Table 5:** Process Efficiency or Apparent Recovery (R_A_) (%): Oral Fluid samples (1 mL) spiked in Quantisal^®^ oral fluid device and extracted by SPE cartridge. R_A(Q)_ (%) and R_A(Q)(n)_ (%).

	R_A(Q)_ (%)	RA_(Q)(n)_ (%)	R_A(Q)_ (%)	RA_(Q)(n)_ (%)	R_A(Q)_ (%)	RA_(Q)(n)_ (%)	R_A(Q)_ (%)	RA_(Q)(n)_ (%)	R_A(Q)_ (%)	RA_(Q)(n)_ (%)	R_A(Q)_ (%)	R_A(Q)_ (%)
Nominal Conc. (ng/mL)	EME	EME_n_ (EME_d3)	CUS	CUS_n_ (EME_d3)	TRO	TRO_n_ (COC_d3)	COC	COC_n_ (COC_d3)	t-CIN	t-CIN_n_ (COC_d3)	COC-d3	EME-d3
10	nd	nd	nd	nd	25%	64%	41%	107%	48%	127%	38%	nd
CV% (n = 3)	-	-	-	-	12.7%	3.8%	11.0%	0.7%	5.0%	7.3%	10.7%	-
20	nd	nd	nd	nd	19–20.2%	63–64.3% (▪)	29.3–31% (▪)	93.7–100% (▪)	29.6–34% (▪)	94.7–112% (▪)	31–31.3% (▪)	nd
CV% (n = 6)	-	-	-	-	5.3	3.5	6.5%	3.4%	8.4%	6.5%	8.6%	-
100	nd	nd	nd	nd	34%	73%	44%	94%	49%	104.4%	47%	nd
CV% (n = 3)	-	-	-	-	-	-	-	-	-	-	-	-
500	12.2%	48%	44.1%	-	-	-	105	114%	190%	159%	90%	28.9%
CV% (n = 3)	8.5%	7.2%	13.3%	-	-	-	14%	3.8%	16.0%	8.4%	17%	6.4%

R_A(Q)_(%) = (absolute area C_Q_/absolute area A) × 100; C_Q_: Spiked OF sample in Quantisal^®^ OF device before extraction; A: neat standard. RA_(Q)_(n)(%) = area (C_Q_/IS-d3)/area(A/IS-d3) × 100; Subscript (n) meaning recuperations IS deuterated normalized. Percentages (%) results are the average of three OF samples spiked at each concentration and extracted by SPE and three neat standards injected. (▪) Percentages (%) results are the average of three OF samples spiked at each concentration and extracted by SPE and three neat standards injected and repeated in two days.

**Table 6:** Extraction recovery or extraction efficiency (RE) (%): Oral Fluid samples (1 mL) spiked in Quantisal^®^ oral fluid device and extracted by SPE cartridge.

	R_E(Q)_ (%)	R_E(Q)(n)_ (%)	R_E(Q)_ (%)	R_E(Q)(n)_ (%)	R_E(Q)_ (%)	R_E(Q)(n)_ (%)	R_E(Q)_ (%)	R_E(Q)(n)_ (%)	R_E(Q)_ (%)	R_E(Q)(n)_ (%)	R_E(Q)_ (%)	R_E(Q)_ (%)
Nominal Conc. (ng/mL)	EME	EME_n_ (EME_d3)	CUS	CUS_n_ (EME_d3)	TRO	TRO_n_ (COC_d3)	COC	COC_n_ (COC_d3)	t-CIN	t-CIN_n_ (COC_d3)	COC-d3	EME-d3
10	nd	nd	nd	nd	24%	70%	35%	104%	33%	98%	34%	nd
CV% (n = 3)	-	-	-	-	11.1%	2.7%	9.3%	0.6%	4.4%	9.5%	11%	-
20	nd	nd	nd	nd	18–20.1% (▪)	69%	25–29% (▪)	94–98% (▪)	20–25% (▪)	78–86% (▪)	26–29% (▪)	nd
CV% (n = 3)	-	-	-	-	3.9%	3.4%	5.22%	2.6%	4.2%	4.5%	5.5%	-
100	nd	nd	nd	nd	32.0%	77.0%	40%	95%	35%	77%	42%	nd
n = 1	-	-	-	-	-	-	-	-	-	-	-	-
500	9.6%	57.3%	33.5%	-	-	-	104%	106%	129	132	98.9%	19.6%
CV% (n = 3)	6.0%	5.8%	46.2%	-	-	-	4.0%	2.5%	8.6%	6.7%	5.28	7.5%

RE(Q)(%) = (absolute area CQ/absolute area BQ) × 100; CQ: Spiked OF sample in Quantisal^®^ device before extraction; BQ: Spiked OF sample in blank Quantisal^®^ device after extraction. RE(Q)(n)(%) = area (CQ/IS-d3)/area (BQ/IS-d3) × 100; Subscript (n) meaning recuperations IS deuterated normalized; nd: not detectable; Percentages (%) results are the average of three OF samples spiked at each concentration and extracted by SPE and three neat standards injected. (▪) Percentages (%) results are the average of three OF samples spiked at each concentration and extracted by SPE and three neat standards injected and repeated in two days.

**Table 7:** Extraction Recovery or Extraction Efficiency (R_E_) (%): Oral Fluid samples (1 mL) taken by passive drooling (spitting) and extracted by SPE cartridge.

	R_E_(%)	R_E_(n) (%)	R_E_(%)	R_E_(n) (%)	R_E_(%)	R_E_(n) (%)	R_E_(n) (%)	R_E_(%)	R_E_(n) (%)	R_E_(%)	R_E_(n) (%)	R_E_(%)	R_E_(%)
Nominal Conc. (ng/mL)	EME	EME_n_ (EME_d3)	CUS	CUS_n_ (EME_d3)	TRO	TRO_n_ (EME_d3)	TRO_n_ (COC_d3)	COC	COC_n_ (COC_d3)	t-CIN	t-CIN_n_ (COC_d3)	COC-d3	EME-d3
50	60.0%	108.0%	53.0%	92.0%	-	-	-	46.0%	119.0%	26.2%	68.0%	39%	56.0%
CV% (n = 3)	6.5%	5.7%	34.0%	39.0%	-	-	-	2.5%	2.0%	4.8%	3.5%	4.0%	7.0%
500	37	74%	36%	70%	47%	93%	54%	63%	73%	64%	74%	87%	50%
CV% (n = 3)	7.1%	5.0%%	14.3%	7.1%	15.0%	10.8%	12.0%	4.0%	0.3%	2.9%	1.5%	4.1%	7.3%

RE (%) = (absolute area C/absolute area B) × 100; C: Spiked OF sample before extraction; B: Spiked OF sample after extraction. RE(n) (%) = (absolute area C/area IS)/(absolute area B/area IS) × 100; Subscript (n) meaning recuperations IS deuterated normalized; Percentages (%) results are the average of three OF samples spiked at each concentration and extracted by SPE and three neat standards injected.

**Table 8:** Limit of detection (+) and limit of quantification (++).

Nominal Conc.	EME		CUS		TRO		COC		t-CIN	
	OF	Q^®^	OF	Q^®^	OF	Q^®^	OF	Q^®^	OF	Q^®^
2.5 ng/mL	nd	nd	nd	nd	nd	nd	nd	nd	nd	nd
5 ng/mL	+	nd	nd	nd	+	+	+	+	+	nd
10 ng/mL	++	nd	nd	nd	++	++	++	++	++	+
20 ng/mL	-	nd	+	nd	-	-	-	-	-	++
50 ng/mL	-	nd	++	nd	-	-	-	-	-	-
100 ng/mL	-	nd	-	nd	-	-	-	-	-	-
500 ng/mL	-	++	-	++	-	-	-	-	-	-

OF: Oral Fluid samples spiked with analytes and extracted in SPE cartridge. Q^®^: Quantisal^®^ oral fluid device spiked with analytes and extracted in SPE cartridge, nd: not detectable.

**Table 9:** Cases studies: Simultaneous samples of OF (collected by drooling and using the Quantisal^®^ (device) were taken at 60 min and 120 min after the ingestion of a cup of coca tea.

**Volunteer 1**						
	**HYG**	**CUS**	**EME**	**TRO**	**COC**	**t-CIN**
OF-60 min	0.28	0.43	0.85	nd	11.94	0.95
Q-60 min	nd	0.08	0.08	nd	1.14	0.07
OF-120 min	0.11	0.05	0.40	nd	1.11	0.13
Q-120 min	nd	nd	nd	nd	0.28	nd
**Volunteer 2**						
	**HYG**	**CUS**	**EME**	**TRO**	**COC**	**t-CIN**
OF-60 min	0.49	0.08	1.66	nd	5.20	0.34
Q-60 min	0.13	0.04	0.27	nd	1.93	0.06
OF-120 min	0.40	0.04	1.73	nd	0.24	0.04
Q-120 min	nd	nd	0.11	nd	0.08	nd

Relation: analyte area/external standard area (SKF); nd: not detectable (area of each analyte is less than the signal-to-noise ratio < 3); OF: Oral Fluid samples (1 mL) taken by passive drooling (spitting) and extracted by SPE cartridge.

**Table 10:** Retention time and ions selected for monitorization.

Group Name	Start Time (min)	Ion Mass-To Charge
HYG	5.00	42; **84**; 141
EME; EME-d3; CUS; TRO	7.50	(82; 94; **96**); (**99**; 85); (**84**; 42; 209); (**124**; 82; 245)
COC; COC-d3; SKF	12.50	(**182**; 198; 303); (**185**; 306); (**86**; 99)
t-CIN	15.10	**96**; 103; 182; 329

Ion quantifier in bold.

## Data Availability

The data that support the findings of this study are available from the corresponding author upon request.

## Supplementary Materials

The following supporting information can be downloaded at: https://www.mdpi.com/1420-3049/29/3/592/s1, Table S1: Matrix effect equations, Table S2: Recovery equations, Table S3: Process Efficiency and Extraction Recovery equations.
