# Supplementary material for: Evaluation of an Oral Fluid Collection Device and a Solid-Phase Extraction Method for the Determination of Coca Leaf Alkaloids by Gas Chromatography–Mass Spectrometry
Source: Molecules. 2024 Jan 25;29(3):592. doi: 10.3390/molecules29030592 (PMC11154435; doi:10.3390/molecules29030592)
Supplement: Supplementary file 1 [file molecules-29-00592-s001.zip › molecules-2771338-supplementary.pdf]

Supplementary Materials

Evaluation of an Oral Fluid Collection Device and a Solid-Phase Extraction Method for the Determination of Coca Leaf Alkaloids by Gas Chromatography–Mass Spectrometry

Pamela Cabarcos-Fernández [1,†], Ivan Álvarez-Freire [1,†], Nelida Cristina Rubio [1], Ana Maria Bermejo-Barrera [1], Antonio Moreda-Piñeiro [2], Ines Sánchez-Sellero [1] and Maria Jesus Tabernero-Duque [1,*]

[1] Forensic Toxicology Service. Forensic Sciences Institute, Faculty of Medicine, Universidade de Santiago de Compostela, Rúa de San Francisco, s/n, 15782 Santiago de Compostela, Spain; pamela.cabarcos@usc.es (P.C.-F.); ivan.alvarez@usc.es (I.Á.-F.); cristinarubio2@gmail.com (N.C.R.); anamaria.bermejo@usc.es (A.M.B.-B.); ines.sanchez.sellero@usc.es (I.S.-S.)

[2] Trace Element, Spectroscopy and Speciation Group (GETEE), Institute of Materials iMATUS, Department of Analytical Chemistry, Nutrition and Bromatology, Faculty of Chemistry, Universidade de Santiago de Compostela, Avenida das Ciencias, s/n, 15782 Santiago de Compostela, Spain; antonio.moreda@usc.es

* Correspondence: mj.tabernero@usc.es

† These authors contributed equally to this work.

Table S1. Matrix effect equations.

Matrix Effect equation	$ME(\%) = \left(\frac{B}{A} - 1\right) \times 100$
Matrix Effect equation with normalized areas (deuterated internal standard)	$ME_{(n)}(\%) = \left(\frac{B}{A} - 1\right) \times 100$
Matrix Effect equation using Quantisal® device	$ME_{Q}(\%) = \left(\frac{B_Q}{A} - 1\right) \times 100$
Matrix Effect equation with normalized areas using Quantisal® device	$ME_{Q\,(n)}(\%) = \left(\frac{B_Q}{A} - 1\right) \times 100$

Table S2. Recovery equations.

$R_{(x)}$	[x $area_C$/x $area_B$] × 100 (.)
$R_{(n)}$(x/x-d3)	[x $area_C$/x-d3]/[x $area_B$/x-d3] × 100 (..)

x: EME, CUS, TRO, COC, t-CIN.; x-d3: EME-d3, COC-d3; (.) Compound area obtained from Study Design C and B (absolute areas); (..) Compound area obtained from Study Design C and B (normalized areas with deuterated).

Table S3. Process Efficiency and Extraction Recovery equations.

Process Efficiency or Apparent Recovery (R_A and $R_{A(Q)}$)	Extraction Recovery or Extraction Efficiency (R_E and $R_{E(Q)}$)
R_A (%) = (absolute area C/absolute area A) × 100	R_E (%) = (absolute area C/absolute area B) × 100
$R_{A(Q)}$ (%) = (absolute area C_Q/absolute area A) × 100	$R_{E(Q)}$ (%) = (absolute area C_Q/absolute area B_Q) × 100
Normalized areas (IS)	
$R_{A(n)}$ (%) = area (C/IS-d3)/area (A/IS-d3) × 100	$R_{E(n)}$ (%) = area (C/IS-d3)/area (B/IS-d3) × 100
$R_{A(Q)(n)}$ (%) = area (C_Q/IS-d3)/area (A/IS-d3) × 100	$R_{E(Q)(n)}$ (%) = area (C_Q/IS-d3)/area (B_Q/IS-d3) × 100
